# Current Approaches in the Multimodal Management of Asthma in Adolescents—From Pharmacology to Personalized Therapy

**DOI:** 10.3390/biomedicines11092429

**Published:** 2023-08-30

**Authors:** Vasile Valeriu Lupu, Elena Jechel, Silvia Fotea, Ionela Daniela Morariu, Iuliana Magdalena Starcea, Alice Azoicai, Adriana Mocanu, Elena Cristina Mitrofan, Ancuta Lupu, Dragos Munteanu, Minerva Codruta Badescu, Magdalena Cuciureanu, Ileana Ioniuc

**Affiliations:** 1 Department of Pediatrics, “Grigore T. Popa” University of Medicine and Pharmacy, 700115 Iasi, Romaniaelena.jechel@yahoo.com (E.J.);; 2 Clinical Medical Department, Faculty of Medicine and Pharmacy, “Dunarea de Jos” University of Galati, 800008 Galati, Romania; 3 Faculty of Pharmacy, “Grigore T. Popa” University of Medicine and Pharmacy, 700115 Iasi, Romania; 4 CF Clinical Hospital, 700506 Iasi, Romania; 5 Faculty of Medicine, “Grigore T. Popa” University of Medicine and Pharmacy, 700115 Iasi, Romania; 6 Department of Pharmacology, “Grigore T. Popa” University of Medicine and Pharmacy, 700115 Iasi, Romania

**Keywords:** teenager, asthma, eosinophils, interleukin, multimodal therapy

## Abstract

Asthma and adolescence are two sensitive points and are difficult to manage when they coexist. The first is a chronic respiratory condition, with frequent onset in early childhood (between 3 and 5 years), which can improve or worsen with age. Adolescence is the period between childhood and adulthood (12–19 years), marked by various internal and external conflicts and a limited capacity to understand and accept any aspect that is delimited by the pattern of the social circle (of the entourage) frequented by the individual. Therefore, the clinician is faced with multiple attempts regarding the management of asthma encountered during the adolescent period, starting from the individualization of the therapy to the control of compliance (which depends equally on the adverse reactions, quality of life offered and support of the close circle) and the social integration of the subject, communication probably having a more important role in the monitoring and evolution of the condition than the preference for a certain therapeutic scheme. Current statistics draw attention to the increase in morbidity and mortality among children with bronchial asthma, an aspect demonstrated by the numerous hospitalizations recorded, due either to an escalation in the severity of this pathology or to faulty management. The purpose of this article is to review the delicate aspects in terms of controlling symptoms and maintaining a high quality of life among teenagers.

## 1. Introduction

Asthma among adolescents represents a challenge for health systems worldwide. The World Health Organization has estimated that approximately 300 million people currently have asthma worldwide (which is expected to increase to 400 million in the coming years), while the latest updates on adolescent asthma state that there are currently approximately 4.136.845 patients in the 11–21 age group (2022) [1,2]. In terms of the symptom prevalence, Ahluwalia and Elwan, in a study from Saudi Arabia (northern border), reported rates of 30.3% for lifetime wheezing, 16.8% for current wheezing (past 12 months) and 19% for exertion-induced wheezing in the previous 12 months. At the same time, high rates of bronchial asthma were highlighted in industrial areas (13.9%) compared to non-industrial areas (8%) [3]. The distribution by sex shows the more frequent diagnosis of asthma among boys (34.4%) as opposed to girls (6.1%), with a predominance of active cases among them (64% versus 20%, respectively), which are often accompanied by wheezing after exercise physical (34.4% versus 12.2%, respectively) [4].

Asthma includes respiratory symptoms caused by hyperreactivity to stimuli, such as wheezing, difficult breathing, expiratory dyspnea, a feeling of tightness in the chest and coughing, having an undulating evolution, marked by frequent relapses and remissions, dependent on environmental factors (physical exercises, smoke, pollen, mold, pollution, microbes, weather changes, strong emotions) [5,6]. It often occurs in association with other allergies (due to the common pathogenic mechanism mediated by IgE), such as allergic rhinitis, conjunctivitis, atopic dermatitis and food allergies, and also with non-allergic disorders, including obesity, gastroesophageal reflux and psychiatric disorders [5]. Respiratory symptoms unresponsive to therapy (to be distinguished from uncontrolled asthma, defined by poor symptom control and/or frequent exacerbations requiring hospitalization) should draw the clinician’s attention for reevaluation to rule out not only cystic fibrosis (CF), non-CF bronchiectasis, immunodeficiency, primary ciliary dyskinesia, bacterial bronchitis or bronchiolitis obliterans, foreign bodies and vocal cord dysfunction, but also psychological problems [7,8]. For children over 6 years and adolescents, four major clinical phenotypes have been described: early-onset allergic asthma, moderate-to-severe early-onset allergic asthma, late-onset nonallergic eosinophilic asthma and late-onset nonallergic noneosinophilic asthma [9].

## 2. Epidemiology

The clearest orientation of the prevalence of asthma in children around the world was outlined by the International Study of Asthma and Allergies in Childhood (ISAAC), which showed significant geographic variations. Thus, the IIIa phase of the study (carried out between 2000 and 2003) ranked the English-speaking countries and Latin American countries in the first places in terms of the diagnosis rate of asthma, the opposite poles being Africa, the Indian subcontinent and the eastern Mediterranean, regions where asthma is less frequently detected, but in more severe forms [1]. The prevalence of chronic asthma in childhood increases inversely proportional to income (relative to the federal poverty threshold (FPL)); thus, it is 8.2% among families with incomes above 200% of the FPL, and 12.2% among those with incomes below 100% of the FPL. In terms of race/ethnicity differences, Black children have a higher overall incidence of asthma [6]. Boys seem to be more prone than girls to exhibit early-onset asthma and pulmonary flow impairment, with the exception of FVC, regardless of ethnicity [10].

In calculating the prevalence, the degrees of underdiagnosis and overdiagnosis must also be taken into account, the quality of life being directly proportionally influenced by these due to incorrectly administered treatment. Aaron et al. have outlined, based on their own studies and from the literature, the potential causes that lead to diagnostic errors, among which we can mention the underreporting of symptoms to the family doctor due to not only the poor perception of the airflow limit and the poverty of respiratory symptoms (31%), diagnostic sensitivity, poor spirometry (sensitivity of 29%, positive predictive value of 77% and negative predictive value of 53%) and low socioeconomic status, but also the impossibility of using objective pulmonary function tests at the time of diagnosis (which leads to an increase in false diagnoses of asthma between 48% and 54%), sustained remission (found in 12% of the study subjects, previously tested positive for asthma and mentioned in the literature as being between 68% and 25%, increasing with the early onset of symptoms) and obesity (rate of error in diagnosis being similar to that of the non-obese population, although other authors put it between 25% and 41%) [11].

Two studies carried out between December 2015 and April 2016 and January and June 2017 aimed at validating the asthma risk factor score (ARFS) with the help of questionnaires focused on socio-demographic characteristics, including age, sex, region, the number of rooms and number of people living in the house, the level of education for both parents (quantified by the number of years of education), the family history of asthma and other known risk factors for asthma (the heating system used inside the house, the history of recurrent otitis, the humidity inside the house, entering the community, etc.) managed to imagine a predictive index for asthma based on the child’s exposure to toxic substances [12].

## 3. Pathogenesis

The National Asthma and Education and Prevention Program Expert Panel Report 3 defines asthma as “a chronic inflammatory disorder of the airways in which many cells and cellular elements play a role: in particular, mast cells, eosinophils, neutrophils (especially in sudden onset, fatal ex-acerbations, occupational asthma, and patients who smoke), T lymphocytes, macrophages, and epithelial cells” [13]. The cause of bronchial asthma is not fully known, but the risk factors (including genetic ones, viral respiratory infections in infants, atopy, changes in the microbiome, abnormalities of vitamin D metabolism) and gene–environment interactions (pollution, stress, exposure to chemicals) play important roles in the pathogenesis of asthma [14,15]. Several studies have shown that the offspring of asthmatic parents, especially on the maternal line, present an increased risk of developing allergies and bronchial asthma due to the vertical transmission of maternal bacteria that influence fetal immune programming [16].

There are two phases of asthma exacerbation: early and late. The pathological process is initiated by IgE antibodies sensitized to certain triggering factors in the environment and released by plasma cells, which then bind to mast cells and basophils with high affinity, causing degranulation and the release of histamine, prostaglandins and leukotrienes, a mechanism that causes the intermittent obstruction of the flow of air. Th2 lymphocytes maintain the process by producing interleukins (IL-4, IL-5 and IL-13—markers of remodeling, fibrosis and hyperplasia) and GM-CSF, supporting inflammation. The late phase represents the concentration at the lung level not only of eosinophils, basophils, neutrophils and T helper and memory cells that maintain bronchoconstriction and inflammation, but also of mast cells that play a role in attracting late-phase reactants to inflamed sites [6,17]. Eosinophilic inflammation, an excess of mucus, and the remodeling of large airways determined a thickened reticular basement membrane and increased airway smooth muscle only in 1/3 of fatal asthma cases, and exclusively in large airways (in correlation with the duration of asthma) [18].

Thus, Barrios RJ. et al., bring to the fore, through a centralization of studies from the last decades, two physiopathological mechanisms based on Th2 action, subdividing the obstructive process according to not only the type of hypersensitivity response (1 versus 4) and the interaction with the targeted receptors (immunoglobulin receptors, leukotrienes or the α chain of the IL-4 receptor), but also the involved interleukins (IL-4, IL-5, IL-9, IL-13). The role played by IL-4 is also emphasized in the production of immunoglobulin E (IgE) antibodies, the target of which is the immune cells that they sensitize for a subsequent encounter with the pathogen, at which point degranulation and the release of inflammatory molecules occur with both direct involvement (amplification of hypersensitivity, glycoprotein production and eosinophilia, similar to leukotrienes) and indirect involvement by stimulating mast cell maturation and IgE secretion [19,20]. Mast cell degranulation occurs as a result of the cross-link formed between two IgE molecules and the causative antigen, which, together with eosinophils activated by means of IL-5, maintain the inflammatory process by means of cytokines [21]. At the opposite pole of the immune mechanism are regulatory T lymphocytes (Treg) that influence the expressions of CD 25, the transcription factor forkhead box P3, transforming growth factor beta (TGF-β) and interleukin 10 (IL-10), components that play a role in modulating the immune tolerance. Beyond these, the regulatory B cell population is also being studied for activity in human subjects with asthma [22]. An essential role in the physiopathogenesis of asthma is also played by the endothelial growth factor, involved in inducing the proliferation of the respiratory vasculature, which has the effect of narrowing the lumen [20].

**Figure 1:**
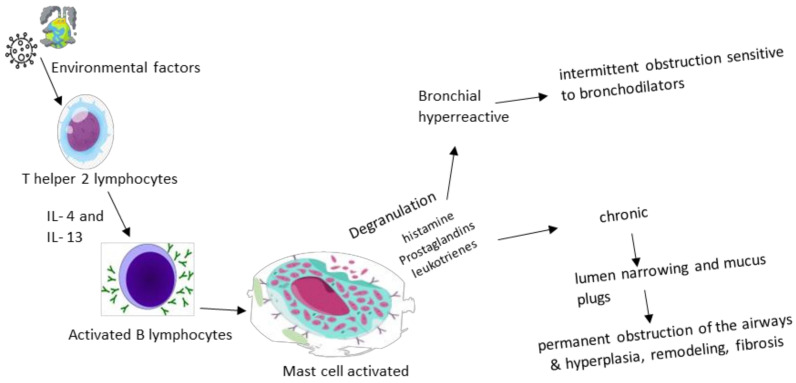
Physiopathological cascade in asthma.

Despite the previous considerations, the current research contradicts the hypothesis of the sterility of the lower airways, an aspect that equally affects the entire range of lung pathologies delimited in our study, especially asthma. With reference to it, Sullivan A. et al. discuss the disruption of bacterial diversity in the lungs of asthmatics, which is characterized by the intensification of the presence of microorganisms such as *Haemophilus*, *Neisseria*, *Moraxella* and *Staphylococcus*, doubled by the decrease in *Streptococcus* species with *Veillonella*, *Faecalibacterium*, *Lachospira* and *Rothia*, disturbances that can still be registered from early childhood, as they are considered risk factors for the subsequent development of asthma or sensitivity to allergens. The circadian rhythm also seems to influence the respiratory microbiota, an aspect objectified by the study of the Bmal 1 gene at the level of the bronchiolar epithelium, which modulates the activity of neutrophils and the host’s response to pathogens, such as *Streptococcus pneumoniae* [23,24,25]. Collaborating with the data presented above, Figure 1 highlights the physiopathological cascade underlying the acute and chronic symptoms found in bronchial asthma.

## 4. Diagnosis

The diagnosis depends on the complexity of the case and the age of the patient, being accessible for the child with characteristic symptoms and cooperative and responding to therapy, and complex for the small child with recurrent wheezing. Asthma is mainly diagnosed clinically when symptoms can be reversed via the administration of a short-acting β2-agonist (SABA), in the absence of another disorder that can cause airflow obstruction.

Gaillard et al. outlined an asthma diagnostic protocol (Table 1) based on the medical history and clinical examination of the patient, to which is added at least one abnormal objective test among spirometry, bronchodilator reversibility testing (BDR), the fraction of exhaled nitric oxide (FENO), the peak expiratory flow variability (PEFR) test and direct and indirect bronchial challenge tests.

Chest X-ray does not change the clinical approach and therapeutic decision in acute asthma crisis, being able to indicate, in the case of SpO_2_ 92% or fever, pathologies such as pneumonia, atelectasis or pneumothorax. Chest X-ray can help exclude some differential diagnoses, such as respiratory infections, foreign body inhalation or congenital diseases (cardiac, lobar emphysema) [27]. Airway narrowing can also be assessed using CT imaging, single-photon emission computed tomography (SPECT), positron emission tomography (PET), magnetic resonance imaging (MRI) and optical coherence tomography (OCT), a new technique that involves the examination of the airways at the time of bronchoscopy and provides insight into the structure of their walls and the mechanical properties with an impact on their narrowing [28].

The asthma severity rating is calculated based on the lung score and oxygen saturation measured via pulse oximetry (Table 2).

## 5. Treatment

For a correct orientation regarding therapy and monitoring, asthma can be subdivided into type 2 (high) and non-type 2 (low) endotypes based on their underlying inflammatory pathways [9].

Medications used to control background symptoms and asthma exacerbations in adolescent patients include inhaled corticosteroids (ICSs), short-acting inhaled bronchodilators (SABAs), long-acting β2-adrenergic receptor agonists (LABAs), long-acting muscarinic receptor antagonists, leukotriene receptors (LTRAs) and, for more serious forms of the disease, specific monoclonal antibodies (IgE (Omalizumab), IL-5 (Mepolizumab, Reslizumab, Benralizumab) and IL-4/IL-13 (Dupilumab)) [5,9,30]. Monoclonal antibodies have the clinical effect of increasing FEV_1_ and quality of life, along with decreasing the need for inhaled corticosteroids and hospitalization. It should also be mentioned that Omalizumab can be administered after the age of 6 years, while Mepolizumab, Benralizumab and Dupilumab can be given after 12 years, and Reslizumab after 18 years [31,32].

Currently, the treatment of the adolescent patient with asthma comprises two stages: the initial treatment, instituted at the first evaluation of the patient, and the subsequent treatment guided by the evolution of the disease. The first treatment regimen is chosen according to the presence and frequency of day/night symptoms, the limitation of physical activity and the risk of exacerbations. Afterwards, it is based on the patient’s response to the administered treatment plan, asthma being classified as controlled, partially controlled or uncontrolled [33]. The choice of their optimal way of administration is made depending on the clinical form of asthma (detailed in Table 3).

Due to the risk of overestimating therapy adherence (72% in the case of nurses and 85% in the case of doctors), various methods of its objective evaluation have been outlined. Among them, we mention the self-report questionnaires (Morisky Scale and MARS-A), the tracking of prescription data, the weighing of the inhaler container, the use of therapy under direct observation and electronic monitoring devices (EMDs) for inhalers [35,36].

Depending on the type of asthma, there are various biomarkers used to evaluate the adherence to therapy, essentially for adolescent monitoring. For type 2-high asthma, potential biomarkers could be serum allergen-specific IgE (SIgE), fractional exhaled nitric oxide (FeNO), the blood eosinophile count, sputum, bronchoalveolar lavage (BAL) or a bronchial biopsy, as well as some cytokines (IL-4, IL-5 and IL-13) or some cytokines specific to the innate immune system (IL-25, IL-33 and TSLP). In contrast, the diagnosis for type 2-low asthma is more challenging. In this case, a diagnosis could be made based on a high neutrophil count in the patient’s sputum or a paucigranulocytic phenotype, with normal-ranged type 2 asthma markers and normal-ranged non-type 2 cytokines (IL-8 and IL-17) [15,27]. Other potential biomarkers include TNF-α and IFN-γ; both of these cytokines contribute to the progression of Th2-low asthma, as well as IL-6 and C-reactive protein, which have been linked with severe asthma [37]. The combined use of biomarkers is recommended to increase the specificity of the results [38].

Comorbidities that affect asthma control can include rhinosinusitis, obesity, gastroesophageal reflux disease, obstructive apnea, psychological disorders as well as medications (angiotensin-converting receptor blockers—ACE-, B-blockers, aspirin and other NSAIDs) [31]. In addition to this, it is important to know the main means of action of the drugs used in practice, the evolutions of which have as a landmark not only the physiopathological cascade encountered in the development of the condition, but also the challenges that clinicians may face in the long-term management of asthmatic patients, particularly children and adolescents, regardless of the choice of an optimal treatment scheme.

Being one of the basic drugs both in the treatment of acute asthma exacerbations and in the long term, systemic or inhaled corticosteroids must be carefully evaluated in terms of the risk/benefit ratio, their over-administration being burdened by increased mortality and adverse effects, such as osteopenia, osteoporosis, muscle atrophy and dyspepsia, impacts on the cardiovascular function, growth curve or visual acuity, the increased incidence of diabetes, obesity, infections and psychological manifestations (depression, anxiety, sleep disorders) as well as the suppression of the adrenal function, manifestations that partially overlap with the aspects related to the non-pharmacological management of patients, developed below. The harmful effects seem to be directly correlated with the frequency of use in the therapeutic scheme, their cumulative nature being still under research [39]. To reduce the adverse effects of the treatment, we discuss the findings of Murphy KR. et al. regarding corticotherapy administered to children younger than/equal to 5 years of age. They demonstrated the effectiveness of nebulization both in acute attacks and for the purpose of maintenance, without, however, identifying a difference between continuous versus intermittent administration [40].

The physiopathological stage targeted by corticotherapy is represented by the production of pro-inflammatory mediators, the stimulation of chemotaxis, adhesion molecules and the antigen–receptor interaction, a stage that it mitigates through its anti-inflammatory role exercised through transrepression. At the molecular level, we note the reduction in eosinophilia, the increase in mRNA degradation, the synthesis of anti-inflammatory proteins and the reduction in vascular permeability with the consequent reduction in liquid extravasation and the stopping of the remodeling process [39,41]. However, not all corticosteroids are similar in terms of effectiveness. Daley-Yates P. et al. exposed the differences between fluticasone furoate, fluticasone propionate and inhaled budesonide, noting the clear superiority of the former [42].

The effectiveness of corticosteroid therapy is influenced by the non-pharmacological conditions to which the patient is exposed, namely, the adherence to therapy and correct inhalation technique, continued exposure to allergenic stimuli in the environment (which can induce corticosteroid resistance mediated by IL-2 and IL-4), associated comorbidities as well as genetic variability (e.g., T-box 21 variants, Fc fragment of the IgE II receptor, histone deacetylase 1, dual-specificity phosphatase 1). Thus, a series of predictive factors of the response to corticotherapy have been outlined over time, which, in the pediatric population, are represented by the pulmonary function at the time of the initiation of therapy, inflammatory markers, the gene functionality or the degree of sensitization to allergens [41].

Therapy based on systemic/inhaled corticosteroids has aroused the interest of researchers regarding the possibility of the similar use of magnesium in refractory bronchial asthma, and specifically moderate/severe bronchiolitis. However, no statistical results were observed to encourage this practice [43,44].

Frequently used in association with corticotherapy, Salbutamol, a short-acting β-agonist, is characterized by a bioavailability of approximately 50% due to the effect of the first hepatic passage. However, it is not without speculation regarding the impact played by its administration on asthmatic mortality, partly due to the possibility of stimulating the pro-inflammatory effect through repeated doses, thus inducing exacerbation. The blood distribution curve after the administration of Salbutamol slowly ascends, an aspect explained from the perspective of its initial action at the level of the bronchial muscles, following that the plasma increase is detected after about 2 h, with a variable half-life depending on the method of administration (of up to 5 h). After binding to the specific receptors (belonging to the family of receptors coupled to the G protein), the conversion of ATP into cyclic AMP occurs, with the triggering of a cascade that has the final result of inhibiting the contraction of the bronchial muscles, in parallel with the effect exerted on the release of mast cell-specific hypersensitivity mediators. Most of the side effects, such as anxiety, tremors, sweating and tachycardia, are due to the complicated binding with the β1 receptor [45,46]. The negative effects of short-term β2-agonist monotherapy were also emphasized by Quint JK. et al., these being associated with the escalation in the risk of exacerbation independent of background therapy [47]. Considering the above, the simultaneous administration in fixed doses of agonists and steroids (e.g., Budesonide–-Formoterol) has been proven to be effective not only at reducing hospitalizations due to exacerbations, but also at improving the quality of life from the perspective of the more facile administration of medication by means of a single inhaler [48,49,50]. In uncontrolled forms of asthma, Virchow JC. et al., demonstrated promising results objectivized by the improvement in the pulmonary function, doubled by the reduction in exacerbation episodes, following the introduction of triple therapy administered with the help of a common inhaler, consisting of corticosteroids, long-acting β2-agonists and antagonists of muscarinic receptors [51].

Therefore, another therapeutic class used in the control of pediatric asthma is represented by muscarinic receptor agonists (anticholinergic). These exert influence through their action on the M1/M2/M3 receptors that modulate the neuronal and non-neuronal signals of acetylcholine, the tone of the respiratory muscles (reducing bronchoconstriction), the mucous secretion of the glands as well as inflammation or remodeling. With regard to the method of administration, one can opt for monotherapy or combined therapy with β-agonists (e.g., Tiotropium/Olodaterol, Aclidinium/Formoterol, Glicopyronium/Indacaterol), the combined preparation form, as well as the long-acting one, recording promising results in the control of asthmatic exacerbations in children over 2 years of age and of long-term symptoms. It is also worth mentioning the superiority of monotherapy with β2-agonists, compared to anticholinergics, when opting for this [52,53,54]. D’Amato M. et al., notes the effectiveness of long-acting muscarinic antagonists, especially among patients who show intolerance to the administration of β-agonists, who have atypical pharmacogenic profiles and in nocturnal asthma [55]. Tiotropium bromide has proven its clinical utility in the treatment of asthma in children and adolescents, its administration improving the lung function, possibly depending on the phenotype. The safety profile was also evaluated, with Dusser D. et al., exposing the main adverse effects of Tiotropium (aggravation of asthma, decrease in maximum expiratory flow or infections in the upper respiratory sphere), which, however, were not reported with an increased incidence compared to the placebo group, rarely doubled by consequences such as dry mouth or urinary retention, arguing thus for the increased tolerability of the drug independent of the ethnic factor or age [56,57].

Having as the main representatives Montelukast, Zafirlukast, Pranlukast or Zileuton, the asthma medications that act at the level of leukotriene receptors with the aim of suppressing production and antagonizing inflammation are divided, depending on the therapeutic target, into G protein-coupled receptor antagonists or inhibitors of 5-lipoxygenase, with action at the level of leukotrienes B4, C4, D4 and E4 [53]. Montelukast, a selective antagonist of the D4 receptor, has proven its effectiveness in bronchial asthma compared to placebos, its administration reducing the need for corticotherapy in controlled forms of asthma. Presenting a peak of action approximately two and a half hours post-administration and at a half-life of 3–4 h, the therapeutic dose in pediatrics is 4 mg/day (2–5 years)–5 mg/day (6–14 years), without the need for adjustment in cases of impaired renal or hepatic function (due to excretion mainly through the biliary tract, except in severe cases). Also, the adequate efficacy and safety profile (the toxic dose far exceeds the authorized upper limit) are certified by mild adverse effects, such as headache, abdominal pain, skin rashes, angioedema and arthralgia, although Özata E. et al. also identify hallucinatory manifestations [58,59,60,61,62]. Similarly, Zafirlukast has proven efficacy and safety in the chronic treatment of pediatric asthma at a dose of 10 mg twice a day [63]. Despite all this, leukotriene antagonists have proven to be inferior in practice to corticotherapy/β-agonists, although the pulmonary effects reach statistical significance, their use being usually reserved for supplementing the therapeutic protocol with the aim of reducing the dose of corticosteroids or in exercise-induced asthma [64].

With relatively little therapeutic experience, partly due to the recent introduction in management protocols, biological agents target individual stages of the physio-pathological cascade, an aspect from which arises the need to study and know the phenotypic particularities of asthma in order to choose the appropriate preparation. The best-known biological agents are Omalizumab (which acts on IgE, reducing the immune response and, consequently, allergic symptoms), Dupilumab (which blocks the IL-4 and IL-13 pathways), Mepolizumab, Reslizumab and Benralizumab (which targets the IL-5 pathway, thus decreasing eosinophilic proliferation) [53]. Antibiotics from the macrolide class (Azithromycin, Clarithromycin) can be added to their support, which, in addition to their anti-inflammatory and antimicrobial roles, have modulatory effects on adhesion molecules and the neutrophil function, and also have a prophylactic function in asthmatic exacerbations caused by respiratory infections with *Chlamydia* or *Mycoplasma pneumoniae* [65]. However, the administration of antibiotics both in the acute phase of asthma and in chronic treatment remains controversial. Other experts in the medical field emphasize the importance of eliminating the unjustified administration of antibiotics, currently estimated to be approximately 7%. This can be performed most easily by outlining some guidelines intended to orient the clinician as to the right moment and the type of antibiotic that can be useful to the patient. The inappropriate use of antibiotics is most frequently encountered in the hospital environment, leading over time to an increase in the risk of resistance to them and to the precipitation of intestinal dysbiosis. The two manifestations lead to a decrease in the ability to respond to subsequent infections that require antibiotic treatment and the favoring of colonization with pathogenic microorganisms, which increase the possibility of the association of various comorbidities [66,67,68,69].

Summarizing the above, the differences from a therapeutic and pharmacological point of view between different age groups reside mainly in the therapeutic classes proven to be effective and safe among them. Thus, in early childhood, the main therapeutic modality is aimed at steroids. Other therapeutic strategies include short-acting inhaled bronchodilators (SABAs), long-acting β2-adrenergic receptor agonists (LABAs) and long-acting muscarinic receptor antagonists and leukotriene receptors (LTRAs). With advancing age, the current guidelines recommend the introduction of monoclonal antibodies to the therapeutic scheme. This introduction is performed successively, having as essential age steps the ages of 6 years (Omalizumab), 12 years (Mepolizumab, Benralizumab, Dupilumab) and 18 years (Reslizumab). At the opposite pole, the treatment of adults aims at the same broad lines; the difference lies not only in a greater variety of monoclonal antibodies that can not only be addressed in therapy, but also in the need to adjust doses according to comorbidities and chronic therapy associated with advanced age. From a pharmacological point of view, although the means of action of the substances are varied, they do not have different administration implications depending on age. The primary difference is the adaptation of the dose and combinations according to body weight/comorbidities/severity, as well as the knowledge of and strict adherence to the parameters of efficiency and safety (especially with regard to modern monoclonal antibody therapies) dictated by the current studies, with statistical relevance.

## 6. Challenges in Management

The chronic nature of asthma, the morbidity and mortality associated with the disease and the high cost of care have determined over time the development of community programs aimed at improving the quality of life of adolescents with bronchial asthma through collaboration with external organizations, institutions and government agencies, with the aims of educating the population and the provision of personalized services, based on personal needs, in specially designed clinics (for example, the asthma management program for children in Kansas City—KC CAMP). The benefits of KC-CAMP are clear, with data showing dramatic decreases in emergency department visits (87%), hospitalizations (85%) and costs of asthma care [70].

Asthma, a chronic and “chameleon” condition, which sometimes strikes in the midst of the lull period, is all the more difficult to manage when it exists/appears during the adolescent period due to the multitude of factors that can intervene individually or in sum in the same individual, escalating the therapeutic challenge with the aim of obtaining and maintaining a satisfactory quality of life (Table 4). Inadequate symptom control is often the consequence of modifiable factors, such as the misdiagnosis of various phenotypes, nonadherence to medications, inadequate inhalation technique, persistent environmental exposures and psychosocial factors [7].

### 6.1. Pulmonary Function

Pulmonary damage, in addition to other associated comorbidities, can be considered a constant in the life of patients with asthma, increasing the risk of COPD in adulthood and negatively impacting the quality of life. Record lung function at the time of diagnosis, 3–6 months after starting treatment, and periodically thereafter (at least once every 1–2 years or more often in patients at risk and in those with severe asthma) should be performed to identify progressive decline [34]. Apart from aspects related to quality of life, it is proven that the pulmonary function in childhood is a positive predictor of asthma remission or persistence: 54% of boys and 70% of girls with an FEV_1/_FVC ratio over 90% go into remission before early adulthood, while less than 10% of people with an initial FEV_1/_FVC ratio of less than 80% reach this point [71]. Numerous studies that have focused on the role of eosinophils in the pathogenesis of asthma have concluded that their increased levels in the blood can predict a lower increase in the lung function (FEV_1_ and FVC) in adolescents with asthma and bronchial hyper-receptivity [72]. Also, the impact is also influenced through the gut–lung axis; however, recently, the hypothesis regarding the sterility of the lower respiratory tract has been dismantled. The inflammation that underlies atopic asthma and seems to determine the severity of airway obstruction is therefore attributed, in part, to the different compositions of the microbiota between asthmatic and healthy individuals [73]. Last but not least, obesity causes significant changes in the mechanics of the lungs and chest, with changes in the breathing pattern through the growth of lung tissue, without changes in the airways [74].

Regarding asthmatic exacerbations, no long-term association with decreased lung function has been identified, returning to pre-exacerbation values after a period of several months in most cases [75]. Some patients still retain a degree of pulmonary insufficiency, resistant to treatment, an aspect that can be attributed to the remodeling of the airways [76]. Although considered to have a bronchoconstrictive effect similar to viral infections or emotions, physical exercises performed with prior preparation (such as walking, jogging, cycling, swimming) are safe and beneficial for patients with asthma, by increasing both oxygen and the ventilatory effort that trains smooth muscle stretching favoring bronchodilation and maintaining the caliber of the airways, as well as through the anti-inflammatory effects exerted [77,78]. Another argument in support of the practice of sports is the existence of the overdiagnosis of exercise-induced bronchoconstriction (EBI), encountered when doctors base their diagnosis only on the history and presentation, without confirming the diagnosis through an objective test (only 8% of 117 children who presented with exertional dyspnea also fulfilled the criteria for EBI). Other diagnoses assigned after excluding EBI at a respiratory specialist clinic were vocal cord dysfunction, poor physical condition, habitual cough and even the absence of any abnormality [79].

### 6.2. Gender

There are multiple hypotheses intended to explain gender differences in asthma symptoms, from an exaggerated perception of bronchial obstruction encountered in women compared to men for the same value to a lower inspiratory muscle strength, as well as hyper-increased bronchial reactivity in women and more frequent mistakes regarding the use of inhaler devices [80].

Although it is known that asthma is more common among boys, this incidence seems to reverse during adolescence when, possibly due to the hormonal storm, the pathology becomes more often identified among girls. Thus, the beneficial effects of androgens on the pulmonary function and symptom control were found, in contrast to the weak harmful effects of estradiol on the pulmonary function in adolescents with asthma [81]. Sex chromosomes also play an important role in the severity of asthma through their influence on the production of sex hormones in the reproductive organs, an example being Klinefelter syndrome, in which the balance is tipped in a negative direction by the association between the supernumerary X chromosome and the relatively low level of testosterone. One of the female X chromosomes is putatively inactivated (15% of the genes on the X chromosome are thought to avoid this mechanism), and the reactivation of immune-related genes is associated with the disproportionate, sex-dependent immune response and, implicitly, with the progression of the disease [82].

At the cellular level, women have greater numbers of B cells and plasma cells with a role in the production of IgE antibodies that bind to FcεRI with high affinity found on mast cells and basophils, determining, upon re-exposure, the release of mediators that contribute to immediate hypersensitivity reactions and bronchoconstriction. Estradiol has also been shown to promote the induction of regulatory B cells and enhance immunoglobulin production [83].

Regarding the female sex, we must also discuss the changes that the body goes through during stages such as menstruation (due to estrogen and progesterone fluctuations, an aspect that causes the peak expiratory flow to decrease), the impact of contraceptive use by suppressing the endogenous fluctuations of sexual steroids (a highly debated aspect at the moment; there are both studies showing a beneficial effect by reducing the symptoms and incidence of asthma, as well as studies denying it for normal and overweight girls, but not for underweight ones) and pregnancy among teenagers [84,85]. During the premenstrual phase, there is an increase in sputum eosinophils and FENO compared to the postmenstrual phase, as well as greater sensitivity to aspirin [84]. Poor control of maternal asthma may be associated with an increased risk of preterm birth, low birth weight, congenital malformations, perinatal death, neonatal hypoglycemia (due to a high dose of beta-agonists in the last 48 h before birth), childhood asthma or even fetal hypoxia due to a small reduction in the mother’s oxygen level. Severe asthma attacks (occurring in ~10% of cases) may represent an indication for caesarean section. It should be mentioned that there is also the risk of anaphylaxis, in the case of women with asthma, when taking oxytocin to induce labor [86].

### 6.3. Asthma Phenotype

After excluding modifiable factors such as adherence and adverse reactions, adolescents whose disease is not well controlled with high-dose medications should be suspected of having a difficult asthma phenotype.

Among the challenges regarding the various asthma phenotypes, the still unknown mechanism of resistance to corticosteroids encountered in eosinophilic asthma (expressed by persistent airflow obstruction and chronic rhinosinusitis with nasal polyps that precedes asthma in most cases) is worthy of consideration. AERD (a subset of the late-onset phenotype that presents both chronic rhinosinusitis with nasal polyps and cyclooxygenase (COX)-1 inhibitor-induced respiratory reactions) and smoking-related asthma increase oxidative stress, neutrophil and macrophage activation and sensitivity to allergens, not necessarily because of the difficult therapy, but rather because of the management of the smoking incidence among teenagers [37].

Neutrophilic, obese asthma is a distinct and difficult-to-manage phenotype that is more common in women (due to increased leptin secretion) than in men. Clinically, it presents with lower lung function and a poor response to corticosteroids compared to non-obese women and men with the same phenotype [86]. In contrast, the paucigranulocytic phenotype of asthma (38–52% of patients) has been associated with a higher body mass index (BMI), younger age, better lung function and the achievement of adequate control with lower doses of inhaled corticosteroids [38].

### 6.4. Compliance with Therapy

The greatest challenge regarding the therapeutic management of asthma among adolescent patients is the communication barrier encountered in some cases due to communication through parents, coming from disadvantaged backgrounds, doctor phobia or the patient’s own non-cooperation, both in childhood, as well as in adolescence, the period in which developmental, psychosocial and environmental changes occur, with cognitive functioning passing from concrete to abstract thinking. Ciprandi et al. evaluated the adherence to therapy through two questionnaires (the Morisky medication adherence scale (MMAS-8) and the TAI), the results showing a poor adherence found in those with severe asthma (MMAS-8: 34.4; TAI: 11.5%) and better but still unsatisfactory adherence in those with well-controlled asthma (MMAS-8: 44%; and TAI: only 20%) [87]. The risk of asthma exacerbation was 21–68% lower for children who had good adherence to control medication compared to those who were less adherent [36].

One of the causes that influences the adherence to therapy is represented by the correct choice and use of inhalation devices, starting from the premise that drugs are effective only if they reach the site of action. The way of choosing and using inhalers must thus represent a key point, both in the priorities of practitioners and patients. The main methods of the administration of inhalation medication are either via nebulization or a metered dose with spacers (MDI). Among these, the latter seems to be more effective at improving the lung function, respiratory rate and therapeutic adherence. In this sense, Normansell R. et al. notes better results in terms of the administration technique among children using MDI. However, we emphasize the need for the individualized monitoring and adjustment of the patient’s inhalation technique, regardless of the age group they belong to. It is also necessary to train the medical staff and create some guidelines intended to guide the clinician towards an appropriate intervention when the attribution of an improper inhalation technique by the patient is observed. Patient education is necessary at the time of diagnosis, at regular intervals and if the device is changed, and this should include the functions of the substances administered with the help of the inhaler and the patient’s satisfaction documented due to the rapid onset of action that determines the immediate improvement in symptoms, when SABA is administered, although regular use is associated with poor outcomes (three or more canisters of SABA per year are associated with increased hospital attendance, while more than 12 per year are associated with the risk of asthma death even in patients with minimal symptoms) [34,88,89,90,91].

Social anxiety disorder (SAD), characterized by the fear of social exposure, a narrow circle of friends, anxiety, depression, poor academic performance and the avoidance of interpersonal relationships, is even more common among adolescents with asthma compared to healthy ones, thus leading to the decrease in compliance with therapy, possibly due to the reluctance to administer their medication in front of others so as not to feel different and isolated from their colleagues [85]. Another cause of the nonadherence (primary) to therapy is represented by depressive symptoms in the caregivers of patients with asthma, such as compromised memory, lack of concentration, the inability to complete tasks and fatigue, drawing attention to the negative impact they have regarding the procurement of medicines from the pharmacy for the child [92].

### 6.5. Associated Comorbidities

There are probable links between obesity and asthma, but the causal relationship is incompletely elucidated. Overweight or obesity, frequently encountered in adolescence, are risk factors for the development of asthma, increasing the diagnosis rate by up to 50%, proportional to the BMI values. The age group most affected is between 7 and 11 years, suggesting that the onset of puberty may represent a time of particularly high risk for obesity-related asthma, especially in girls. In boys, the risk becomes higher after the age of 12 years, reiterating the impact of gender in pediatric asthma [93]. The quality of life of patients with asthma and obesity is significantly lower in the long term due to the increased prevalence of the metabolic syndrome, insulin resistance, dyslipidemia (especially through the decrease in the high-density lipoprotein fraction), psychosocial problems, upper respiratory tract disorders, obstructive sleep apnea syndrome and gastroesophageal reflux among these patients [74]. In the long term, obesity, osteoporosis, anxiety and depression are more common in women with asthma compared to men [86].

Gastroesophageal reflux disease (GERD) is another pathology often associated with asthma, an aspect that increases the chance of patients manifesting GERD by up to 2.86 times, being not only an important step in the therapeutic course of asthmatics, but also proof of the need to address possible complications when we face resistance to therapy [94]. Thakkar et al. examined the prevalence of GERD, estimated to be approximately 60% for adults, analyzing 20 articles in which it was documented via pH testing, contrast imaging, impedance, esophagogastroduodenoscopy and questionnaires based on symptoms or diagnostic codes, and noting that there is a possible association between GERD and bronchial asthma in children, but this is incompletely researched due to the lack of longitudinal studies [95]. A treatment strategy for patients with GERD and asthma initially includes proton-pump inhibitors (PPIs), followed by frequent reassessments for 6 months and the continued administration of PPIs in the same way for those who do not have frequent exacerbations and where the adequate suppression of acid is objectivized with the help of the pH probe or by impedance, while, in the absence of improvement, it is recommended to escalate the dose of PPIs split into two doses to associate a prokinetic for a period of up to 1 year or to resort to surgical intervention (fundoplication) if the secretion pH is above the current standards (i.e., pH < 4 in distal esophagus for <5% of the time) [96,97].

Chronic rhinosinusitis (CRS), the form associated or not with nasal polyps, has been reported as a frequent comorbidity of asthma, which shares the same endotypes and increases its exacerbation crises [98]. According to studies, any asthmatic patient with an unpredictable response to adequate treatment must be investigated to exclude the concomitant presence of sinusitis, and treated with antibiotic therapy, nasal deobstruction with saline solution and the administration of topical steroids, in case of its discovery, even if it does not exacerbate the symptoms background in the present [99].

Allergic rhinitis is a frequent chronic condition, classified as seasonal or perennial, depending on the environment from which the triggering allergen originates, with an increasing prevalence that affects 10–20% of the world’s population and up to 80–90% of asthma patients, having a negative impact on their quality of life. Clinically, there is serous rhinorrhea in increased or possibly posterior amounts, nasal congestion, sneezing and itching of the nose/throat, itching/watering of the eyes, headache and hyposmia. Anti-IgE treatment can be considered in patients with severe asthma who are sensitized to perennial aeroallergens, and the response is often satisfactory [100,101,102].

Due to its high prevalence, allergic rhinitis can be considered the classic model that highlights the importance of the recognition and adequate management of comorbidities. Thus, as explained by Maio S. et al., and Magnan A. et al., the presence of allergic rhinitis is directly correlated with the increase in the burden of the disease by the association of more severe forms of asthma and the decrease in quality of life. Thus, community medicine, carried out by family doctors and general specialists, must represent the first line in recognizing, raising awareness of and counteracting the negative effects and comorbidities associated with respiratory diseases (in this case, asthma). They must be well informed about the diagnostic and management protocols and involved in the evolutionary courses of their patients. The expected final results are mainly the decrease in the reaction time and the intervention of a specialist, doubled by a prompt, individualized and more accessible approach for the patient. All this leads to a healthier population, with a better quality of life and a greater ability to integrate into social life [103,104].

Obstructive sleep apnea (OSA) and asthma are closely related, forming a vicious circle in which OSA accentuates the severity of asthma through the additional impact brought on by chronic intermittent hypoxia, increased respiratory effort and chronic stress caused by sleep fragmentation, while asthma is a strong risk factor for the development of OSA. In addition, their coexistence can be associated with the previously stated pathologies (rhinitis, gastroesophageal reflux and obesity), the increase in abdominal pressure during periods of OSA contributing to the occurrence of GERD, hyperreactivity and bronchial inflammation. The treatment that can be attempted in order to reduce morbidity, especially when these two pathologies exist simultaneously, is represented by CPAP [101,105].

The paradoxical movement of the vocal cords characterized by their adduction with airflow limitation is another condition that can mimic or worsen asthma, as it is difficult to differentiate due to similar symptoms, such as wheezing, dyspnea and cough, which are associated with stridor and hoarseness. The triggering factors are represented by the conditions that lead to increased laryngeal sensitivity, such as posterior rhinorrhea, gastroesophageal reflux and respiratory infections, but a psychopathological component can also be found in its development, a fact that attests to the important role of the pediatric psychologist within the multidisciplinary committee meeting to discuss the pathology. Diagnosis can be difficult, as the laryngeal exam is normal outside of attacks, as is the flow–volume curve. A noninvasive therapeutic intervention that aims to improve symptoms by educating patients on exercises designed to relax the vocal cords is speech therapy [102,106].

### 6.6. The Psychological and Social Component

Anxiety and depression seem to be a constant both for patients with asthma and for their relatives, an aspect that can affect the evolution of the disease given the fact that the severity of the disease and its control and therapeutic effectiveness are closely related to the psychological integrity of adolescents. To evaluate the psychological status, various questionnaires have been used that have identified an emotional deterioration among the patients [107]. A study conducted by comparing a group of 61 adolescents with asthma and a similar control noted that the academic success and social relations of the adolescents with bronchial asthma were inferior to the healthy controls, suggesting that there is an association between asthma, the degree of victimization and repression anger. The results of the study reiterate the importance of psychosocial assessments and screening in this population, emphasizing the impact determined by psychological factors on the management of adolescents with asthma [108]. In association with the above, forming a vicious circle that endangers the health and development of the patient, we find that the abuse of substances such as marijuana, cannabis, cocaine or alcohol increases in direct proportion to the degree of alteration in the psychological status. Consequently, the American Medical Association (AMA) recommends that adolescents (between 11 and 21 years old) be examined for the consumption of alcohol/drugs and be advised by doctors to avoid the consumption of “tobacco, alcohol, and other abusable substances”, given the risk of its underreporting [109,110].

Another determinant of the development, exacerbation or poor control of bronchial asthma is the regular exposure to tobacco smoke (at least seven cigarettes per day, on average, in the previous week, and 300 cigarettes in the previous year), which predominantly affects non-allergic adolescents and those exposed to maternal smoking during the intrauterine period (increased risk up to eight times in smokers exposed in utero compared to the group of non-exposed non-smokers). The association between active smoking and bronchial asthma was not substantially influenced by the education level, family income, other demographic factors, birth weight, gestational age, health insurance, physical activity levels, family history of asthma, pets, humidifier use, other household characteristics or exposure to ambient pollutants and indoor combustion sources, including second-hand smoke [111]. Similar results were also observed with regard to electronic cigarettes, until recently considered harmless compared to traditional cigarettes, with vaping proving harmful to the respiratory mucosa by affecting the lung function and exacerbating respiratory symptoms [112,113,114]. Therefore, the current “epidemic” of smoking, both classic and vaping, represents a possible source of alteration of the fragile pulmonary balance, especially among asthmatic adolescents, who should be protected as much as possible from joining this vice under the influence of their entourage and from the desire for social validation.

**Figure 2:**
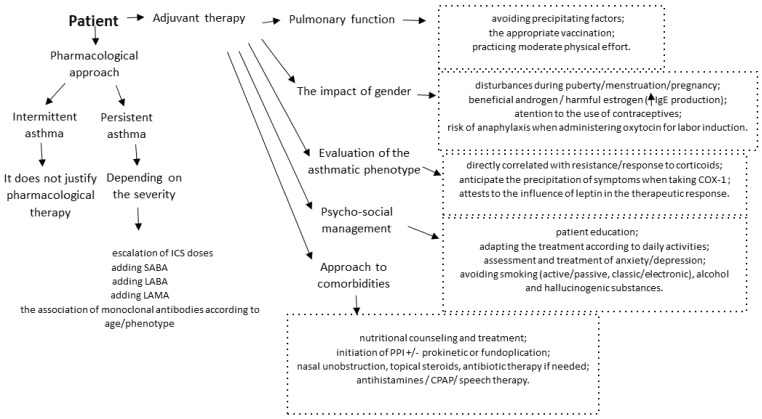
Individualized therapy of the asthmatic adolescent.

It is vital to establish the patient’s ability to take part in daily activities, as it has been proven that, in most cases, it is necessary to administer a preventive SABA or physical exercises to reduce the bronchoconstriction induced by them. Inhaled corticosteroids in a single dose in subjects who do not benefit from them as a background treatment also offer a protective effect, an aspect not found in the case of those treated regularly with ICSs, in which there is a reduction in the frequency and severity of bronchoconstriction without, however, eliminating the need for acute therapy [115]. Regarding the impact on mortality, studies demonstrate that severe uncontrolled asthma increased the risk of mortality only for patients without a history of psychiatric disease, but there was no significant difference between patients with severe uncontrolled asthma and the general population in patients with a history of psychiatric disease [116]. Summarizing the data stated above, Figure 2 shows the optimal way of therapeutic management of the asthmatic patient. This must be centered on both the pharmacological component and the complementary one, adjuvant therapy.

## 7. Before Closing

The recent pandemic that we went through changed multiple visions in medical practice, from diagnostic criteria (certainty or differential) to therapeutic means, going through a phase of global uncertainty that brought with it the need to limit social interaction and made it difficult to manage chronic conditions, aspects that have negatively affected quality of life. The reduction in healthcare utilization by adolescents with chronic asthma may be explained by changes in risk factors determined by COVID-19, such as air quality through reduced pollution, the indoor environment, physical activity, weight control and medication management. As a respiratory condition, asthma was initially considered a risk factor for the development of SARS-CoV2 infection, based on the premise that excessive mucus secretions from the bronchi, epithelial lesions, airway obstruction and local immunosuppression caused by inhaled corticosteroids create an environment conducive to its development, aspects that were later refuted in most studies centered on children. Contrary to expectations, statistical data showed lower rates of COVID-19 in adolescents and children compared to adults, possibly due to an association between the lower expression of angiotensin-converting enzyme 2 (ACE2) receptors in the respiratory epithelium and more frequent exposure to a high variety of pathogens, leading to immunity to common coronaviruses [117,118]. Also, SARS-CoV-2 has not been incriminated as an important factor in exacerbating wheezing or asthma in allergic children, an aspect that seems to be valid for the childhood asthma–obesity phenotype. Chest radiographic changes were generally less pronounced than in adults, the most characteristic pattern described as being “ground glass” opacifications in 62.3% of CT scans, mostly in the lower (44.4%) and unilateral (53.4%) lobes, the consolidation with the surrounding halo sign being considered a pathognomonic sign described in 9.4% of pediatric patients [119].

Adolescent vaccination against COVID-19 is also a sensitive topic in medical practice, depending equally on parental concerns about vaccine safety/side effects and the reluctance of medical professionals to share recommendations for increasing vaccination adherence, which leads to hesitation, refusal and delay in obtaining parental consent, although the strong association between adolescent and parental immunization is proven [120,121]. Basch et al., drew attention to the impact of social media on spreading vaccination misinformation, pointing out that one of the most dangerous aspects was the display of a parody/meme of an adverse reaction, even before the vaccine was distributed to the public, due to the discrepancy between high levels of digital literacy and the relatively low health literacy among young people, the result being the limitation of the ability to critically evaluate online content [122]. Regarding adverse reactions and the vaccine response among adolescents, the literature shows that neutralizing antibodies were higher after vaccination compared to adults, and adverse reactions were mainly mild-to-moderate transient reactions, including pain, flushing and swelling at the injection site, and nausea, diarrhea, fatigue, fever and headache, without serious adverse events [123]. Rarely, cases of myocarditis or pericarditis, cardiac arrhythmias leading to death, acute chest pain and elevated troponin have been reported [124,125]. Bronchial asthma is therefore not a contraindication for vaccination against COVID-19, with the mention of its administration in the stage of clinical remission of symptoms and not in acute asthma attacks (wheezing, coughing, breathing difficulties, feeling of chest tightness and other symptoms), especially when glucocorticoids are used systemically (oral and intravenous). The vaccine can also be administered during anti-IgE monoclonal antibody therapy and allergen-specific immunotherapy, but it should not be administered on the same day [123].

## 8. Conclusions

Asthma is a complex pathology that is under continuous study both in terms of the diagnostic means and therapeutic strategies. Being a chronic pathology with a peak incidence in childhood, it leaves a mark on patients’ lives in all its stages, one of the most delicate being adolescence. Thus, it is important to know that any adolescent who does not respond to standard asthma medication and has recurrent pulmonary symptoms may fall under particular asthmatic phenotypes or have associated comorbidities, such as sleep apnea, obesity, gastroesophageal reflux disease, allergic rhinitis, rhinosinusitis or vocal cord dysfunction, which form a vicious circle with negative repercussions for therapeutic control. Worthy of reiteration is the positive impact of physical activity on the control of bronchoconstriction encountered in this pathology, but the reduction in bronchial inflammation, in contrast to the hypotheses that promote the negative effect of EBI, has proven to be overdiagnosed. The psychological component and social integration are not to be ignored either, having a say both in terms of the patient’s quality of life as well as the adherence to therapy, which can also be disrupted due to the fear of being different and isolated from their circle of friends, in addition to aspects related to the degree of the procurement of the medication, the nature of the inhaler device, the false impression that short-acting drugs offer adequate control over the symptoms and the influence of anxiety and depression encountered both among subjects and caregivers. We also emphasize the impact of gender on the frequency of asthma exacerbations, boys falling into a more protected area during adolescence, unlike girls. Finally, we draw attention to the need to develop longitudinal and observational studies on the behavior of asthmatics in association with various pathologies that can affect the population on a large scale, as well as on drug interactions between the therapy of these conditions and asthmatic therapies, given the multitude of associated comorbidities with asthma that can lead to cardiovascular, digestive or neurological damage with the automatic involvement of the association of different therapeutic classes that can potentiate or inhibit each other, thereby influencing the physiopathological balance and homeostasis of the body.

## Figures and Tables

**Table 1:** Asthma diagnostic protocol (adapted from European Respiratory Society clinical practice guidelines for the diagnosis of asthma in children aged 5–16 years) [26].

Criteria	Remarks	Recommendation
Symptoms include wheeze, cough and breathing difficulty	A history of recurrent wheeze—the most important symptom of asthma; Chronic cough (for >4 weeks) as the only symptom—further investigations to exclude differential diagnoses should be considered.	Strong recommendation against the intervention.
Improvement in symptoms following preventer medication	Only in symptomatic children with abnormal spirometry and negative bronchodilator response; The objective tests spirometry and, if indicated, BDR testing should be repeated after 4–8 weeks.	Conditional recommendation against the intervention, based on clinical experience.
Spirometry testing	Diagnosis: FEV_1_/FVC below the LLN or <80% or; An FEV_1_ < LLN or <80% predicted. A normal spirometry result does not exclude asthma.	Strong recommendation for the intervention.
BDR testing	Consider an increase in FEV_1_ ≥ 12% and/or ≥200 mL following inhalation of 400 µg of a short-acting β_2_-agonist as diagnostic of asthma. BDR < 12% does not exclude asthma; Consider BDR testing when baseline spirometry is normal if the clinical history is strongly suggestive of asthma.	Strong recommendation for the intervention, based on clinical experience, in all children with FEV_1_ < LLN or <80% pred and/or FEV_1_/FVC < LLN or <80%.
F_eNO_ testing	Diagnosis: F_eNO_ value ≥ 25 ppb in a child with asthma symptoms. A F_eNO_ value < 25 ppb does not exclude asthma.	Strong recommendation for the intervention.
PEFR variability	Other objective tests are preferred, but a PEFR variability test can be considered in healthcare settings lacking other objective tests; If a PEFR variability test is used, the result should be based on 2 weeks of measurements, ideally using electronic peak flow meters; A cut-off of ≥12% in PEFR variability should be considered a positive test. PEFR variability of <12% does not exclude asthma.	Conditional recommendation against the intervention.
Allergy testing (skin-prick tests to aeroallergens and serum total and specific IgE tests)	Overdiagnosis of asthma, particularly in children with other atopic diseases, and underdiagnosis if physicians rely on allergy tests for asthma diagnosis.	Strong recommendation against the intervention.
Direct bronchial challenge testing (methacholine and histamine)	PC_20_ value of ≤ 8 mg/mL^−1^.	Conditional recommendation for the intervention.
Indirect bronchial challenge testing (exercise and mannitol)	A fall in FEV_1_ of > 10% from baseline; Mannitol challenge can be considered as an alternative to exercise challenge. Mannitol challenge should be best avoided in favor of other challenge tests (limited availability in most countries and children often find the test unpleasant).	Conditional recommendation for the intervention.

BDR: bronchodilator reversibility; FEV_1_: forced expiratory volume in 1 s; FVC: forced vital capacity; LLN: lower limit of normal; F_eNO_: exhaled nitric oxide fraction; PEFR: peak expiratory flow rate; PC_20_: provocative concentration of methacholine that results in a 20% drop in FEV_1_.

**Table 2:** Framing the severity of asthma (adapted from Pediatric Asthma: The REGAP consensus) [29].

	SpO_2_ %	Lung Score	Respiratory Rate		Wheezing	Use of Accessory Muscles	Score
			<6 Years	≥6 Years			
			<30	<20	Not	Not	0
Easy	>94	0–3	31–45	21–35	End of exhalation (with stethoscope)	Low	1
Moderate	91–94	4–6	46–60	36–50	Full exhalation (with stethoscope)	Moderate	2
Severe	<91	7–9	>60	>50	Both inspiratory and expiratory (no stethoscope)	Excessive	3

Regarding the lung score, points are received from 0 to 3, depending on the severity, for each of the 3 indices considered.

**Table 3:** Treatment steps in asthma in adolescents (adapted according to GINA 2021) [34].

Intermittent Asthma	It Does Not Require Treatment	
Persistent asthma	Step 1.	
	Preferred reliever: → As needed, low-dose ICS-formoterol	Alternative reliever: → Take ICS whenever SABA is taken
	Step 2.	
	Preferred reliever: → As needed, low-dose ICS-formoterol	Alternative reliever: → Low-dose maintenance ICS
	Step 3.	
	Preferred reliever: → Low-dose maintenance ICS-formoterol	Alternative reliever: → Low-dose maintenance ICS-LABA
	Step 4.	
	Preferred reliever: → Medium-dose maintenance ICS-formoterol	Alternative reliever: → Medium/high-dose maintenance ICS-LABA
	Steps 5.	
	Preferred reliever: → Add-on LAMA → Refer for phenotypic assessment +/− anti-IgE, anti-IL5/5R, anti-IL4R → Consider high-dose ICS-formoterol	Alternative reliever: → Add-on LAMA → Refer for phenotypic assessment +/− anti-IgE, anti-IL5/5R, anti-IL4R → Consider high-dose ICS-LABA

**Table 4:** Challenges in asthma management.

Pulmonary function; Gender; Asthma phenotype; Therapy compliance; Associated comorbidities; The psychological and social component.
